# Open-Label Uncontrolled, Monocentric Study to Evaluate the Efficacy and Safety of the Electromagnetic Field and Negative Pressure in the Treatment of Cellulite

**DOI:** 10.3390/life15071148

**Published:** 2025-07-21

**Authors:** Antonio Scarano, Antonio Calopresti, Salvatore Marafioti, Gianluca Nicolai, Erda Qorri

**Affiliations:** 1 Department of Innovative Technologies in Medicine and Dentistry, University of Chieti-Pescara, 66100 Chieti, Italy; 2 The Hospital Giuseppe Fogliani, 98057 Milazzo, Italy; antoniocalopresti@gmail.com; 3 Azienda Sanitaria Provinciale Reggio Calabria, 89128 Reggio Calabria, Italy; salvatore.marafioti@gmail.com; 4 Dental School, University of Chieti-Pescara, 66100 Chieti, Italy; nicolaigianluca@gmail.com; 5 Odontostomatology Department, Maxillo-Facial Surgery, University di Tor Vergata, 00133 Rome, Italy; 6 Dental School, Albania University, 1001 Tirana, Albania; erda79@yahoo.com

**Keywords:** cellulite, body contouring, skin laxity, skin tightening, electromagnetic field, negative pressure, vacuum

## Abstract

Cellulite is a widespread aesthetical dermatological condition affecting a significant proportion of postpubertal women, characterized by dimpled skin, primarily on the thighs, buttocks, and hips, which has an important psychological impact. Cellulite, also called lipodystrophy or oedematosclerotic panniculitis, causes an aesthetic change in the skin that affects the epidermis, dermis, hypodermis and subcutaneous fat in different ways. The aim of the present prospective study research was to evaluate the efficacy of electromagnetic field and negative pressure in the treatment of cellulite. Methods: A total of 35 women with an average age of 40, ranging from 18 to 50 (mean 32.2 ± 7.48), with a body mass index between 18.5 and 26.9 (mean 22 ± 3.01), were enrolled in this study. The degree of cellulite of the patients was assessed clinically using the Cellulite Severity Scale (CSS) and Nürnberger–Müller classification. All patients received one session per week for a total 12 treatment sessions with Bi-one^®^ LifeTouchTherapy medical device (Expo Italia Srl—Florence—Italy), which generates a combination of vacuum and electromagnetic fields (V-EMF). Total treatment time was approximately 20–30 min per patient. The GAIS score, Cellulite Severity Scale (CSS) and Nürnberger–Müller classification for cellulite was evaluated 1 month after the 12 treatments with LifeTouchTherapy. Results: A statistical difference was recorded in cellulite improvement by visual analog scale (VAS) and global aesthetic improvement scale (GAIS). Conclusions: The results of the present prospective clinical study show the efficacy and safety of Bi-one^®^ LifeTouchTherapy in the treatment of cellulite. Electromagnetic fields combined with negative pressure therapy promote tissue regeneration and reduce fibrosis, which results in visible cosmetic improvements of cellulite.

## 1. Introduction

Cellulite is a frequent aesthetical dermatological condition affecting a significant proportion of postpubertal women, characterized by dimpled skin primarily on the thighs, buttocks, and hips, with an important psychological impact [1].

Cellulite, also known as lipodystrophy or oedematosclerotic panniculitis, causes an aesthetic change in the skin that affects the epidermis, dermis, hypodermis and subcutaneous fat in different ways [2], with an incidence of about 85% to 90% of the post-pubertal female population [3]. This alteration is so widespread in Anglo-Saxon countries that it is defined as a normal and unavoidable skin expression of women in the peritrochanteric area and its adipose layer [3].

Cellulite occurs in women of all races, although it seems to be more common in Caucasian women [4]. The pathophysiology involves factors such as genetic predisposition, hormonal influences, and structural differences in the skin and subcutaneous tissue.

The etiology of cellulite is not yet defined with certainty, although there are two main theories established over the years and corroborated by many authors. The first considers it to be due to a homeostatic alteration of the microcirculation, while the second refers to the different arrangement of collagen fibers in women compared to men. Such differences between men and women have been found in the architecture of the body since birth [4,5]. The oblique orientation in men, as opposed to the perpendicular orientation of fibrous septa in women, contributes to the characteristic appearance of cellulite [5]. Cellulite is commonly perceived as a cosmetic problem. The mechanisms underlying its development remain unclear, but current research indicates a multifactorial origin involving hormonal, structural, and vascular components. In particular, endothelial dysfunction and microcirculation disorders appear to play a significant role. These findings support the idea that cellulite may not be solely a cosmetic issue, but rather a manifestation of broader systemic imbalances and homeostasis disorders [6]. A wide range of therapeutic approaches have been developed to improve the appearance of cellulite. These include laser and radiofrequency treatments, acoustic wave therapy, subcision techniques, vacuum-assisted tissue release, massage, topical and injectable agents.

According to Curri, the primum movens of cellulite is an alteration in the homeostasis of the skin’s microcirculation, a theory shared by several researchers [7]; disorganization of the cutaneous blood microcirculation is reflected in the vasomotion and flowmotion functions of the capillary system and causes increased thickness and sclerosis of the fibrous septa [8]. Estrogen, oxidative stress and inflammation alter the microcirculation with a reduction in lymphatic drainage, causing edema [8]. The concept of vasomotion, i.e., the functionality and peristalsis of the blood vessels, should involve a sequence of 3 to 20 compression and release cycles per minute, which are essential to determine the peripheral thrust on the microcirculation, and which determine the variation of intravascular fluid flows (flowmotion) [9].

According to some authors, impaired microcirculation in the gluteo-femoral area leads to hypoxia, which in turn promotes fibrosis of the subcutaneous connective tissue [10,11]. Tissue hypoxia appears to be related to the increase in glycosaminoglycans (GAGs) in the capillary network attracting fluid and causing edema with consequent congestion of the capillary network, which progressively reduces its functions [12].

In women, the fibrous septa are perpendicular to the skin surface, contributing to increased pressure on the subcutaneous fat towards the interface between the dermis and hypodermis, causing the fat tissue to herniate towards the dermis [4,5,13,14].

In practice, the adipose tissue of the subcutis is pushed towards the dermis, leading to irregular herniations that manifest themselves visually in the form of a ‘quilted blanket’ or ‘orange peel [15,16,17]’. It is assumed that the fibrous shoots correspond to the atrophic areas of the tissue burdened with cellulite, commonly referred to as cellulite holes, while the herniation’s represent the part that has escaped towards the dermis. An magnetic resonance imaging (MRI) study of female patients with cellulite showed the presence of many fibrotic septa perpendicular to the stratum corneum, extending from the dermis to the subcutis, whereas in women without cellulite and in men, they are less numerous [16].

Ultrasonographic analysis confirms this finding, showing that the increased evidence of cellulite is coupled with increased herniations of adipose tissue in the dermis [18]. However, the etiology of cellulite is multifactorial and includes genetic predisposition, gender, hormonal and anatomical factors, lymphatic deficits, microcirculation function and lifestyle [15,19,20,21], which act alone or simultaneously. According to other authors, in addition to the fibrous septa and the microcirculatory deficit, there is an aesthetic problem that affects the positioning and quality of the adipose tissue as well as the loss of skin volume [4,5,8,10,15,22].

Various therapies have been proposed for the treatment of cellulite, characterized by modest, often temporary, results and generally ineffective in the medium term [23].

Electromagnetic fields combined with negative pressure therapy have been shown to be particularly effective in promoting tissue regeneration and reducing fibrosis [24,25]. The rationale for this study stems from the growing interest in non-invasive, multi-modal approaches to cellulite management. While various technologies have been individually studied, there is limited evidence on the combined use of capacitive radiofrequency, variable negative pressure, and electrostimulation. The aim of the present study was to evaluate the efficacy of electromagnetic field and negative pressure in the treatment of cellulite.

## 2. Materials and Methods

### 2.1. Efficacy Evaluation

Patients with cellulite were recruited for this study. A total of 35 women with an average age of 40, ranging from 18 to 50 (mean 32.2 ± 7.48), with a body mass index between 18.5 and 26.9 (mean 22 ± 3.01), were enrolled in this study. The cellulite grade was classified according to a clinical diagnosis of cellulite, with grades I to III according to the classification that ranges from 0 to III, located on the thighs and/or buttocks. None of them were in the menopausal phase. All patients exhibited common characteristics, dimpled skin in the gluteal and posterior thighs. Individuals who had modified their diets, were pregnant, had a history of heavy smoking (≥20 cigarettes per day), had a history of allergic or irritant contact dermatitis of the hands, were affected by systemic diseases, or had diagnosed psychiatric disorders were excluded from participation. The clinical investigation was conducted at the Department of Medical Sciences of the University of Tirana, Albania. The study adhered fully to ethical principles, including the guidelines set forth in the World Medical Association Declaration of Helsinki [26], as well as the additional requirements specified by Albania law.

Each participant provided informed consent prior to undergoing the prescribed procedure.

**Figure 1:**
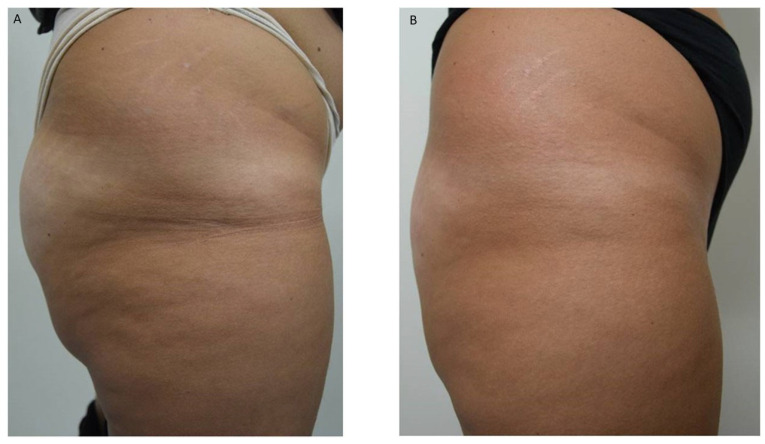
(**A**) Patient with cellulite before and (**B**) after 12 sessions of Bi-one^®^ LifeTouchTherapy weekly.

**Figure 2:**
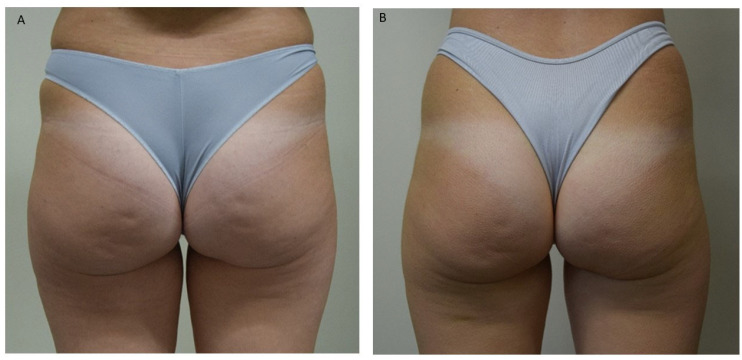
(**A**)—Before treatment. (**B**)—A reduction in the number of dimples is observed.

**Figure 3:**
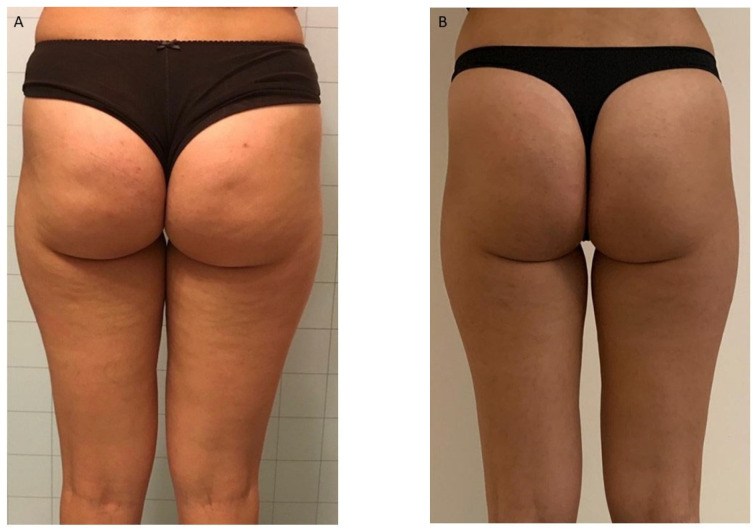
(**A**)—Before treatment. (**B**)—A reduction in the number of dimples and skin laxity is observed.

After anamnestic information collection and physical evaluation, the patients were photographed in the gluteal and trochanter regions. The photographs were taken by the same investigator, with the same camera fixed at the same location. All patients stood at the same distance from the camera, in the standing position with relaxed gluteus muscles, as illustrated in Figures 1–3. All patients were of Caucasian ethnicity, with 41% being classified as Fitzpatrick skin type II and 59% as type III. All participants maintained their regular diet and physical activities throughout the entire study period. The degree of cellulite of the patients was assessed clinically using the Cellulite Severity Scale (CSS) and Nürnberger–Müller classification and was classified as mild with score 1–5, medium with score 6–10 and severe when the score was 11–15. All patients received one session per week for a total 12 treatment sessions with the Bi-one^®^ LifeTouchTherapy medical device (Expo Italia Srl—Florence—Italy), which generates a combination of vacuum and electromagnetic fields (V-EMF), already adopted for cellulite treatment. Total treatment time was approximately 20–30 min per patient. Cellulite improvement was recorded with the visual analog scale (VAS) and global aesthetic improvement scale (GAIS). The GAIS score was evaluated 1 month after the twelve treatments with electromagnetic field and negative pressure (EMF-NP). The EMF-NP is a medical device in Class IIB that produces an electromagnetic field with a variable frequency between 0.5 and 2 MHz with an average intensity of 4 W, and a variable negative pressure between 0.090 and 0.200 millibar; those energies are delivered with one handpiece. The frequency and the intensity of the electromagnetic field are determined by an artificial intelligence installed in the device, reading in real time the feedback from the patient.

The handpiece is covered with a single-use cap in non-cytotoxic PVC. The patient was made to lie on the bed in a prone position, and a thin cosmetic film, Jaluro Gel, Amino Gel (Expo Italia Srl—Florence—Italy) was applied to the patient’s skin for better coupling, and greater fluidity of the movement of the handpiece. The handpiece was made to slide all over the buttock and trochanter, focusing the action on the areas burdened by cellulite and stretch marks.

The efficacy of EMF-NP was evaluated based on the progress observed during follow-up, which was assessed using photographic archives and the reporting of an independent physician who was blinded to the procedure, using the GAIS score. Skin evaluation for irritation was performed using direct visual inspection under standardized lighting conditions, with the aid of a magnifying lens (×2.5) to enhance the detection of subtle changes in skin morphology.

The type of light used was a cold LED source with a color temperature of 5500 K to ensure consistent and non-invasive illumination.

The parameters monitored included erythema, edema, dryness, desquamation, and any signs of discomfort or hypersensitivity.

Observations were conducted immediately after treatment, and subsequently at 24, 48, and 72 h, with a final follow-up at 7 days post-treatment.

Each subject was evaluated four times post-treatment to monitor for any delayed adverse effects. The data were collected prospectively using standardized Case Report Forms (CRFs), which were completed by trained clinical staff during scheduled study visits. All data entries were verified against source documents in accordance with ISO 14155 guidelines [27].

### 2.2. Safety Evaluation

#### 2.2.1. Adverse Events (AEs)

The investigation had to be monitored regularly according to the Standard ISO 14155 and national and local regulations. Case report forms (CRFs) were to be reviewed against source data for adherence to the Clinical Investigation Plan (CIP), as well as for completeness, accuracy, and consistency of the data.

Case report forms were reviewed by the coordinating investigator for omissions, apparent errors, or values requiring further clarification. Findings were entered on data correction forms and referred back to the collaborating investigators for resolution and subsequent correction.

An AE was defined as any untoward medical occurrence, unintended disease or injury, or any untoward clinical signs (including abnormal laboratory findings) in subjects, users, or other persons. For subjects, this definition included events, whether or not related to the investigational device, and those events related to the procedures involved. For users or other persons, this definition was restricted to events related to the investigational device.

#### 2.2.2. Adverse Device Effects

An adverse device effect (ADE) was defined as an AE that was related to the use of the investigational device. This included any AE resulting from insufficiencies or inadequacies in the instructions for use, the deployment, the implantation, the installation, the operation, or any malfunction of the investigational device. In addition, this included any events that were a result of a usage error or intentional misuse.

### 2.3. Statistical Analysis

The study data were collected using a specially designed electronic database and assessed by the Graphpad 8 software package (Prism, San Diego, CA, USA). The descriptive statistical methods considered the median, mean, standard deviation, 95% CI. The Wilcoxon test for categorical data was assessed to calculate the statistical significance among the variables investigated. The linear regression model and Pearson correlation matrices have been applied to intercept the interactions with the independent variables (age, BMI).

A *p*-value < 0.05 was considered significant. The population sample for single-arm analysis was calculated using G*Power software package, GraphPad 9 (Prism, San Diego, CA USA) considering an α error of 0.05 and power (1-β) of 0.80 [H1slope: 0.4; H0slope: 0.0]. The minimum sample size considered a total of 34 patients.

## 3. Results

All patients were photographed at baseline and one month following their final session. Treatments resulted in visible cosmetic improvements, as evidenced by photographic assessments (Figure 1, Figure 2 and Figure 3). The GAIS score for cellulite was evaluated 1 month after the 12 treatments with EMF-NP. The descriptive statistics of the enrolled patients have been summarized in Table 1, Table 2 and Table 3, indicating means, standard deviations, 95%CI, median, 25th, and 75th percentiles.

**Figure 4:**
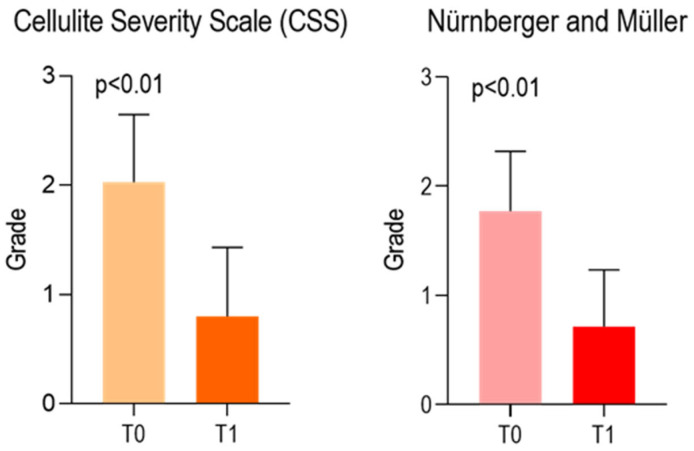
Charts of the Cellulite Severity Scale (CSS) (**left**). The Cellulite Severity Scale (CSS) proposed by Rossi and Vergnanini. The scale is based on a scoring system that evaluates five morphological parameters, each rated from 0 to 3: visible depressions at rest; number of depressions; surface appearance of the skin; degree of skin laxity. Total Score Interpretation: 1–5 → mild cellulite or Grade 1; 6–10 → moderate cellulite or Grade 1; 11–15 → severe cellulite or Grade 1. Nürnberger and Müller Scale (**right**) assessed at T0 and T1 [Wilcoxon test]. Nürnberger and Müller Scale is a qualitative clinical classification system: Grade 0: No visible cellulite, even when the skin is compressed (pinch test);Grade I: No visible cellulite in standing or lying positions, but an orange-peel appearance is evident upon skin compression; Grade II: Cellulite is visible in the standing position but not when lying down; Grade III: Cellulite is clearly visible in both standing and supine positions, with pronounced skin alterations.

**Figure 5:**
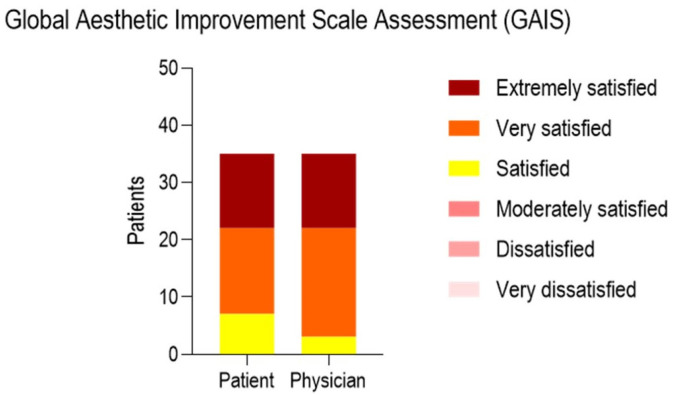
Charts of the Global Aesthetic Improvement Scale Assessment (GAIS) grading submitted to the patients and the physician.

At the baseline (T0), the mean values of CSS and Nürnberger and Müller Scale were, respectively, 2.029 ± 0.6177 and 1.771 ± 0.5470. At T1, the CSS and Nürnberger and Müller Scale were 0.8000 ± 0.6325 and 0.7143 ± 0.5186. A significant difference was observed comparing both scores at T0 and T1. (Figure 4). The Global Aesthetic Improvement Scale Assessment (GAIS) submitted to the patients and physician is presented in Figure 5 and Table 3.

No irritation area was observed either during or after the treatment. There was no presence of pain or side effects recorded, including skin necrosis or alteration of pigmentation. No AEs were observed. During the treatment, immediately after its completion, and at subsequent time points (24, 48, and 72 h), with a final follow-up at 7 days post-treatment, no signs of erythema, edema, dryness, desquamation, or any other indications of discomfort or hypersensitivity were observed. Only a mild transient redness was noted immediately after the end of the treatment, which resolved spontaneously within approximately 10 min.

## 4. Discussion

The outcome of the present study shows a significant visible cosmetic improvement of cellulite after treatment with EMF-NP. A reduction in the degree of cellulite was observed according to CSS and Nürnberger and Müller Scale. These results were confirmed by the patients and the physician using the GAIS score. The linear regression of the interactions between cellulite scales and independent variables did not reveal any statistical significance with respect to body mass index (BMI) and age. However, the limited sample size does not allow for definitive conclusions. Moreover, it should be noted that our sample consisted predominantly of normal-weight patients, with only a few cases of mild overweight, and no obese patients were included in the study. Several studies suggest an association between body mass index (BMI) and the degree of cellulite. The findings indicate that a higher BMI is associated with greater severity of cellulite, although the relationship is not always linear and may be influenced by other factors such as fat distribution, genetics, and lifestyle. However, it is important to emphasize that cellulite can also be present in individuals with a normal BMI, and that BMI alone is not a sufficient indicator to predict its presence or severity [28,29]. Other authors have found a strong positive correlation between the thickness of the adipose tissue fold (which increases with BMI) and the degree of cellulite [30].

Cellulite has great negative psychosocial effects, including anxiety, body dissatisfaction, and decreased quality of life. The aesthetic distress caused by cellulite leads many women to seek treatment, despite the condition being painless without impact on the general health of patients. Many treatment options for cellulite include mechanical stimulation, topical agents, laser therapy, radiofrequency, magnetic field, negative pressure and injectables like collagenase clostridium histolyticum. EMF-NP acts simultaneously on cellulite, alterations, fibrous septa and microcirculation. The electromagnetic field, in its various configurations and deliveries, identified in the literature by different names and acronyms such as CRET, EMF, Marconitherapy, Tecar, capacitive RF, dermal filler, has been shown to be able to reduce cellulite fibrosis [31,32,33,34].

Negative pressure has also shown remarkable efficacy in reducing skin fibrosis due to cellulite [34,35]. The application of magnetic fields combined with negative pressure induces a moderate increase in skin temperature, which does not exceed 39 °C. This controlled thermal elevation has been associated with a reduction in scar tissue fibrosis and the stimulation of neocollagenesis and elastic fiber formation [36].

The fibrous septa present in cellulite causes compression of blood and lymphatic vessels, resulting in hypoxia and edema [8,10,11,12].

Cellulite becomes more evident as the years go by due to reduced collagen synthesis, elastin degradation, and loss of hydration, leading to laxity and atrophy of the skin [37,38,39,40]. Therefore, it cannot be ruled out that cellulite forms at a young age and becomes visible in old age due to reduced skin turgor. A histological study of 70 patients with varying degrees of cellulite documented important morphological changes in the dermis involving the extracellular matrix, collagen fibers, microcirculation and lymphatic vessels [2].

A recent histological study has shown that magnetic fields associated with negative pressure result in an increase in angiogenesis [41]. The magnetic fields cause an overheating of about two degrees that reaches a depth of 10–20 mm, resulting in an increase in blood circulation that lasts up to 30 min after the end of the delivery of the magnetic fields, resulting in an increase of about 15% in hemoglobin saturation, which on average exceeds the value of 98.1 [42]; these results were confirmed by Tashiro et al. [43]. The increased temperature of tissue treated with magnetic fields increases blood flow compared to a group of patients treated with placebo therapy [44].

Magnetic fields have an anti-inflammatory action. In fact an increase in IL10 has been documented in the treated tissues [45]. Similarly, negative pressure has been shown to activate the production of anti-inflammatory cytokines [46].

Magnetic fields and negative pressure have an anti-inflammatory action and reduce fibrosis and improve tissue oxygenation [36] and angiogenesis [41], effects that are particularly useful in the treatment of cellulite. EMF-NP it is useful in reducing fibrosis and increasing vascularization, which is why it has been successfully used in the treatment of stretch marks, scars [24,47] and skin aging [48]. Static magnetic fields exert an anti-inflammatory effect primarily through the modulation of pro-inflammatory and anti-inflammatory cytokine production, as well as through cellular-level changes. A demonstrated that inhomogeneous static magnetic field (SMF) exposure significantly enhanced IL-10 production in both macrophages and lymphocytes. At the same time, it reduced the secretion of pro-inflammatory cytokines such as IL-6, IL-8, and TNF-α [49]. A systematic review conducted by Longano et al. [50] xamined the use of electrophysical agents, including radiofrequency, electric currents, and ultrasound, for management of cellulite. Although radiofrequency was used in 28% of the studies reviewed, the authors noted that the overall methodological quality was low and only a limited number of studies demonstrated strong evidence of efficacy [50]. Vacuum therapy has also been shown to improve skin texture and reduce the appearance of cellulite by enhancing lymphatic drainage and stimulating fibroblast activity. However, its effects are often temporary and dependent on treatment frequency [1]. Electrostimulation, while less commonly used as a primary treatment, has been reported to improve muscle tone and local circulation, contributing to modest improvements in skin appearance. However, its impact on deeper fibrotic structures is limited compared to RF or combined modalities. Compared to these approaches, our protocol combining electromagnetic fields and negative pressure seems to offer synergistic benefits. Unlike many monotherapies, our method acts simultaneously on several aspects of cellulite pathophysiology: fibrous septa, microcirculation, and inflammation [46].

Skin tissues treated with EMF-NP recover the correct values of hydration, sebum and pH and elasticity [24,47]. Cellulite is a multifactorial condition with important psychological and social impacts. Clarifying its pathophysiology is essential for developing effective cellulite management strategies. Dietary and lifestyle interventions also play a role in treating this common aesthetic dermatological condition. Various therapeutic strategies, including mechanical stimulation, topical agents, tissue subcision, dermal filler and injectables, show varying degrees of efficacy. The limits of the present study are a small sample number and the fact that it is not a randomized clinical study. Participants’ lifestyle factors such as diet, physical activity, and stress levels were not controlled or monitored during the study period. These variables could have significantly influenced the results, particularly in the context of cellulite management.

The follow-up period was limited to one month post-treatment. This short duration does not allow for evaluation of the long-term sustainability or potential recurrence of the management effects. In future we intend to perform a comparative randomized clinical study with a larger sample.

## 5. Conclusions

In conclusion, the results of the present clinical observation study show the efficacy and safety of electromagnetic field and negative pressure in cellulite management. Electromagnetic fields combined with negative pressure therapy promoting tissue regeneration and reducing fibrosis resulted in visible cosmetic improvement of cellulite.

## Figures and Tables

**Table 1:** Summary of the Cellulite Severity Scale (CSS) and Nürnberger and Müller Scale assessed at T0 and T1.

**Cellulite Severity Scale (CSS)**		
	T0	T1
*Median*	2.000	1.000
*Mean*	2.029	0.8000
*Std. Deviation*	0.6177	0.6325
*95% CI of mean*	(1.816–2.241)	(0.5827–1.017)
*25% Percentile*	2.0	0.0
*75% Percentile*	2.0	1.0
*Δ mean (T0–T1)*	1.2286 ± 0.5983	
**Nürnberger and Müller Scale**		
	T0	T1
*Median*	2.000	1.000
*Mean*	1.771	0.7143
*Std. Deviation*	0.5470	0.5186
*95% CI of mean*	(1.584–1.959)	(0.5362–0.8924)
*25% Percentile*	1.0	0.0
*75% Percentile*	2.0	1.0
*Δ mean (T0–T1)*	1.0571 ± 0.4815	

**Table 2:** Linear regression of the cellulite scale interactions with independent variables. No significance was detected considering the body mass index (BMI) and age.

**Cellulite Severity Scale**	**BMI**	**Age**
F	0.4032	2.003
DFn, DFd	1, 33	1, 33
*p*-value	0.5298	0.1664
Pearson’s r	−0.1099	0.239
**Nürnberger and Müller**	**BMI**	**Age**
F	0.9702	1.465
DFn, DFd	1, 33	1, 33
*p*-value	0.3318	0.2347
Pearson’s r	−0.1690	0.239

**Table 3:** Grading of Cellulite Severity Scale (CSS) and Nürnberger and Müller Scale assessed at T0 and T1.

**CSS Cellulite Severity Scale**			**Nürnberger and Müller**		
	T0	T1		T0	T1
None (0)	0	6 (29%)	None (0)	0	6 (29%)
Mild (1)	4 (19%)	12 (57%)	Mild (1)	6 (29%)	14 (66%)
Moderate (2)	12 (57%)	3 (14%)	Moderate (2)	13 (62%)	1 (5%)
Severe (3)	5 (24%)	0	Severe (3)	2 (9%)	0

## Data Availability

Data can be obtained from the authors upon reasonable request.
